# Cataphoresis

**Published:** 1897-10

**Authors:** C. L. Hungerford


ARTICLE VII.

### CATAPHORESIS.

BY DR. C. L. HUNGERFORD.

**Read before Kansas State Dental Association.**

I have had some little experience with cataphoresis. It has been fairly satisfactory, from the fact that I have only used it in a certain character of cases. I have found it is extremely satisfactory in those cases where you can bring your electrodes in direct contact with the soft tissues. In other words, it is a method of driving cocaine or any other anesthetic into the tissues very much more rapidly than the normal physiological process of absorption would take it in. If you apply cocaine to the mucous membrane, you find its anesthetic effect is wholly superficial, just upon the bare tissue; by aid of the electric current, which mechanically carries forward the drug, it is driven deeply into the body of the tissue, thereby anesthetizing it. I find cataphoresis is very satisfactory in obtunding the gum where you desire to lance an alveolar abscess in cases where a tooth has been extracted and the remaining socket is very painful to the touch and does not heal readily; and if you have an exposed pulp, you can apply your cataphoresis to that pulp and obtund it in a very short time. For the preparation of sensitive cavities, in obtunding sensitive dentine, I have found that it is very slow and somewhat uncertain.

I myself have been using a storage battery. I have tried the dry cells and I have tried the ordinary street circuit. I find there is very little difference in the effect from either the cell, storage battery, or the street circuit. I think the storage battery is the most satisfactory, the cell battery comes next, and the street circuit is the least satisfactory. I use the street circuit because it is so much more convenient. It is always ready at hand for me, and I have no batteries to keep in order and requiring attention. To those few who live where they can not have the advantages of the street 110-volt circuit, as we call it, then a storage battery can be used, if you have a convenient place to send it to be recharged, and if not, you will be compelled to use the ordinary cell battery.

In using the street circuit, as I use it now, I adopt a method which obviates a serious objection to the 110-voolt current. The mains come in the office here, and I have those run through a rheostat made of coils connected with the main. This is, of course, cut in or cut out, whenever I wish to use it. When I cut that in, the entire current coming to my office forms what is called a short circuit, so that in case the wires become crossed with the arc light system, or in case of a thunderstorm or any accident which may occur to the main circuit, the current will be made to pass through that rheostat, which is not connected at all with the patient. Electricity is very much like a stream of water passing through a pipe; if you put a little hole in the pipe and take a stream off from the pipe in that way, no matter how much pressure there may be upon the large main, it will only increase the pressure or volume of water at the hole to a very slight extent. If I have my patient over here [illustrating on blackboard], I have what I call a "shunt-circuit." I connect my patient with two wires; one of them is immovably fast here, and the other one is capable of passing along a slide, so that I can run in more or less of what we call a shunt, giving more or less current. Then the current passes through the rheostat, it passes through this shunt, through the patient and back to the main circuit, so the dangers from the use of the street circuit are almost entirely done away with.

Then we almost always have little fuse-blocks fastened in the main circuit or fastened in the rheostat, so that they will burn down very rapidly in case any extra amount of current should come across the wire.

Now, that is the current I have been using almost exclusively for cataphoresis. There is one main objection to the ordinary incandescent circuit, and that is because it is irregular. The current as it comes across these wires, instead of being a straight line, as I have marked it here, is rather a zigzag line; in other words, what we call the voltage is rising and lowering all the time on account of the slipping of the belt and slowing down of the dynamo or from the lack of force in the steam engine to drive the dynamo with the requisite speed. Lights, instead of burning with what they call 110-volt pressure, are ordinarily burning with from 90 to 105 or 115 volts. You very rarely find the voltage in a building on a circuit run up to the requisite standard. If it is a private plant, located in a building, the proprietor learns that the life of the lamps is very much lengthened by running with a lower pressure. If the dynamo is run at a very much higher speed, the lamps become very much more incandescent, but will burn out very much more rapidly, so they scarcely ever run them up to the requisite standard; and even then the voltage is irregular, and irregularity in the current is productive of pain to the patient when used in cataphoresis.

The current which comes from a storage battery or primary battery is very much more steady, and thus is much less productive of pain than the street circuit, but the latter, if used with ordinary care, is not apt to produce much pain.

In using a storage battery, it takes about 20 cells. We find we generally get about 2 volts to each cell of the storage battery, and generally 1 to 2 amperes. I have a battery which gives 2½ amperes to each cell and 2½ volts. That is scarcely sufficient to perform cataphoresis unless you can have a complete exposure of the soft tissues. You need 30 to 50 or 60 cells, really, to get the best effects, because you have to get enough pressure to force the current entirely through the body, and if you are supplying it to a very dense tooth, you have an enormous amount of resistance. The resistance is stated to be 50,000 or 60,000 ohms in a tooth. If this is the case, you are going to have such a minimum amount of electricity forced through that tooth that there is no cataphoretic effect obtained at all from the application of the drug, or not enough to be appreciated. You have to have enough current to send through there to produce the effect. It is not pressure that produces the desired effect, it is the quantity of electricity; but you must have sufficient pressure behind it to force it through.

Now, in applying it to a tooth where there is very sensitive dentine and a small cavity, you have to drive it through so much of the body of the tooth-structure that it takes anywhere from 15 minutes to three-quarters of an hour before the cocaine is really carried into the body of the tooth. Therefore, I have discarded cataphoresis in the preparation of sensitive cavities. I have a better, surer and more rapid method.

Now, as I said last night, just as sure as there comes before the profession a method of completely anesthetizing pulps of teeth, dentists are going to get into trouble. They are going to be careless in their manipulations and the pulps of teeth are going to be exposed without either themselves or the patients knowing it, and they are going to pack amalgam or gold fillings into those cavities and the death of the pulp will ensue.

It has been the experience of a great many operators that the pulps of teeth have become dead from the use of cataphoresis; not because of any detrimental effect from the application of the current, not because of any disease, or because of any irritating effect from the cocaine, but simply because the tooth had been so thoroughly anesthetized that the operator has cut right into the body of the pulp, or has approached so nearly to it that it will be made to die from thermal changes, or from irritation from the metal or whatever filling may have been introduced after the cavity was prepared. It is not the electricity, not the cocaine, but simply because, not being warned by the extreme sensitiveness of the cavity as you approach the pulp, you will cut too close to the body of the pulp, and later on you will find it diseased.

Therefore, if operators will use any means of producing anesthesia that is really effective, they must understand the technique of tooth-structure, and be thoroughly familiar with the anatomy and the position of the pulp, and then the most extraordinary caution must be used, because you have nothing to warn you when you are cutting into that pulp. And very frequently we find irregular pulp-chambers, and find some pulps extending up into the little cusps, and are likely to expose one of these and have an abscessed tooth at the end of a few weeks or a month.

Now, as to the nerves and tissues. Everybody knows cocaine will benumb the nervous tissue whenever it comes in contact with it. The cataphoretic effect of the electric current is simply the mechanical forcing of cocaine into the tissues that would not so readily absorb it without it. If I were to attempt to drive a nail into this table by simply laying it point down on the table or pressing on it with my hand, it would take a very long time, and probably never would stick in, but take a hammer and you can force it in very quickly, and that is all the electric current does in cataphoresis. The electric current has nothing to do with the anesthesia. It is not sufficient to produce any irritation to amount to anything. It has been taken by people the world over thousands and thousands of times into the body—as strong a current as we employ for cataphoresis, and has never done any harm. It is not destructive of tissue, and does not produce irritation to any extent. It is employed many times in strong currents for a cautery—for its heating effect.

The only kind of current we can use for cataphoresis is one which goes simply in one direction. If it passes in one direction and then quickly comes back, constituting what we call an alternating current, it drives the cocain in, and when it comes back it drives it o t again. Therefore the alternating current is of no service. There is a current known as the Farady current—that is an induced current which is given off from winding a coil of wire around a magnet, forming a little primary battery. That current is not strong enough to produce electrolysis; it is coming in such an infinitesimally small amount, there is really no carrying in of the substance.

The current employed in cataphoresis is very much like a cyclone passing along a tract of country; anything that lies in the path of that cyclone will be moved and carried forward. Anything that lies in the path of this current is carried along with the current mechanically by the mere force of the current, and carried on in a circular, spiral-like motion into the tissues and along the path of the conductor.

Now I have tried it where I have had exposed pulps, and found I could anesthetize that tooth in from 3 to 5 minutes by the cataphoresis ordinarily very satisfactorily, so I could remove the pulp of the teeth entirely; or perhaps I would make a second application in 3, 4 or 5 minutes after, and remove the contents of the pulp-chamber without causing any pain. I have had very excellent results in applying it to cavities where a tooth has been extracted, using a small pledget of cotton and leaving it in there for 3, 4 or 5 minutes.

I have had very unsatisfactory results with it in applying it to a tooth that was very sensitive, where there was sensitive dentine around the neck of the tooth, where there was a very small, shallow, saucer-shaped cavity and a large body of the tooth-structure to penetrate. The tooth-substance is such an extremely bad conductor it takes the current a long time to work its way through the dentine, and needs a greater amount of current and a greater length of time.

I find great use for it; perhaps on an average of once a week in my office I have occasion to use my outfit. I could not use it any more for obtunding sensitive dentine, though I do not believe that any detrimental or dangerous effects follow.

It might be worth while to follow this by enquiring what kind of solution is the best to use. What is known as a solution that offers absolutely no opposition to the current—in other words, a solution that is a perfect conductor—is the one that is not going to produce any very great effect. You are going to have the current pass readily through that drug, and the result may be that none of the cocaine will be carried in, or only a very small amount. The idea of carrying cocaine into the tissues is based on the principle that there is an obstruction to the current at the point of application, and the greater that obstruction is the more intense will be the effect, and the more deeply will the cocaine be carried into the tissues, but it will not be carried in provided it is a perfectly good conductor of the current. Distilled water is known to be a good non-conductor, and therefore we add it to the cocaine salt (or possibly some other salt) to render it more of a non-conductor. Then guaiacol, or guaio cocaine, as it is called, is much recommended. Guaiacol is simply a preparation of creosote. It is somewhat of a local anesthetic, and a solvent for cocaine, and it is a very poor conductor of the electric current. Therefore cocaine dissolved in guaiacol makes a very excellent application for the purpose of carrying cocaine into the tissues by the electric current.

Ordinary water I find just about as satisfactory, although I find that it varies some as a non-conductor of electricity.

I do not care to go into the subject of electricity, or further into the subject of appliances. Those who live in the city can have an electrician fix them up a rheostat where they can have a little shunt they can take off, and they will find that as satisfactory as anything. Those who have not this current available can get a battery, have it enclosed in a glass case, with a little milliampere meter, or something to show the direction of the current. They are really the most satisfactory for those living outside of the larger cities where they have not the use of a main circuit.

I believe that simply cocaine dissolved in water is just as good an application as any of the others that we can employ. I believe that the danger of its application is not from the cocaine itself or from the current, but it is in the following manipulation after the tooth-structure has been thoroughly and completely anesthetized.

As to the conductor to be used in applying the cocaine, you should employ for your electrodes some kind of metal that is not going to be disintegrated by the electric current. If I use a copper wire to convey the salt, I find there is an electrolysis of that wire. In fact, the electric current can not pass through any substance without producing an electrolitic action, the effect of which is a molecular change of the structure of that body, no matter whether it is a copper wire, or human tissue, or water. If I use a copper wire on my positive pole, the current passing from the positive to the negative, I will find the salts of copper will be driven into that tooth-substance, and the result is the tooth will turn green. The only electrodes which are permissible to use are platinum, in which metal there is no apparent decomposition of the metallic substance of which they are composed. There is simply electrolysis of the liquid itself, or, if any takes place in the metal, it is so infinitesimal that it is not appreciated.

Instead of placing the patients upon the negative electrode, or allowing them to hold it in their hands, I generally give them a sponge to hold against the side of the face, so that instead of passing the electric current through the entire body, I pass it through a small amount of tissue; the patients holding the moistened sponge under the jaw, then applying the positive pole saturated with the water and cocaine into the cavity, direct, or on the inflamed gum where I expect to make an incision. This must be done with the positive pole, as the current passes from the positive to the negative. If you apply the cocaine on the negative pole, you would have absolutely no result—if any thing, the effect would be irritating. It is a well-established fact that the positive pole is hemostatic and will stop the flow of blood; it has a quieting effect, while the negative pole is an irritant and produces increased bleeding in a wound. Whenever you want irritation, you apply the negative pole; whenever a sedative effect is sought, you use the positive pole. So that, in my opinion, there may be some slight sedative effect produced from the application of the positive pole in cataphoresis from the current itself; but the real anesthesia comes from the cocaine.—*Western Dental Journal.*

---

